# 2,3,7,8-Tetrachlorodibenzo-*p*-dioxin (TCDD) and Polychlorinated Biphenyl Coexposure Alters the Expression Profile of MicroRNAs in the Liver Associated with Atherosclerosis

**DOI:** 10.1155/2020/2652756

**Published:** 2020-08-12

**Authors:** Qiuli Shan, Fan Qu, Ningning Chen

**Affiliations:** ^1^College of Biological Science and Technology, University of Jinan, Jinan 250022, China; ^2^State Key Laboratory of Environmental Chemistry and Eco-Toxicology, Research Center for Eco-Environmental Sciences, Chinese Academy of Sciences, Beijing 100085, China

## Abstract

MicroRNAs (miRNAs) are a class of small RNAs that regulate gene expression. 2,3,7,8-Tetrachlorodibenzo-*p*-dioxin (TCDD) and polychlorinated biphenyls (PCBs) are persistent organic pollutants that exist as complex mixtures *in vivo*. When humans are simultaneously exposed to these compounds, the development of atherosclerosis is known to be enhanced. However, the roles of miRNA in TCDD- and PCB-induced atherosclerosis are largely unknown. Therefore, the present study is aimed at elucidating the possible dysregulation of miRNAs in atherogenesis induced by coexposure to TCDD and PCBs. Eight-week-old male ApoE^−/−^ mice were coexposed to TCDD (15 *μ*g/kg) and Aroclor1254 (55 mg/kg, a representative mixture of PCBs) by intraperitoneal injection four times over a 6-week period. Microarray analysis of miRNAs and mRNAs in the liver of ApoE^−/−^ mice with or without TCDD and Aroclor1254 coexposure was performed. We discovered that 68 miRNAs and 1312 mRNAs exhibited significant expression changes in response to TCDD and PCB coexposure and revealed that both changed miRNAs and mRNAs are involved in cardiovascular disease processes. An integrated miRNA-mRNA approach indicated that miRNA-26a-5p, miRNA-193a-3p, and miRNA-30c-5p participated in specific TCDD and Aroclor1254 coresponsive networks which are relevant to the cardiovascular system development and function network. Furthermore, our results also indicated that miRNA-130a-3p and miRNA-376a-3p were novel players in the regulation of TCDD- and Aroclor1254-induced atherosclerosis pathways. In summary, our finding provided new insights into the mechanism of atherosclerosis in response to TCDD and PCB coexposure.

## 1. Introduction

Cardiovascular disease (CVD) has become a major cause of morbidity and mortality in many countries [1]. Atherosclerosis is regarded as the leading cause of CVD, which is present in all humans of advanced age and continues to develop throughout life [2, 3]. Atherosclerosis is a chronic disease of the arterial wall that is characterized by the formation of atherosclerosis plaques on the inner walls of the arterial vessel [4]. Generally, the plaques are composed of the accumulated foam cells containing lipid particles, proliferated vascular smooth muscle cells (VSMCs), and extracellular matrix [5]. Moreover, monolayer cells, macrophages, and lymphocytes are also critical players in the development of plaques [6]. In this context, it is important to mention that lipids have a central role in the pathogenesis of plaques, and the liver is the main tissue which plays important roles in the regulation of lipid metabolism [3].

MicroRNAs (miRNAs) are a family of noncoding, single-stranded, 17–25-nucleotide long RNAs. miRNAs can bind to the complementary site of the target mRNA through its 3′-UTR, thereby regulating protein-coding gene expression at posttranscriptional and translational levels [7]. MicroRNAs regulated diverse biological processes, including cancer and metabolic diseases [8–10]. Recently, several research reports showed that miRNAs also played a vital role in atherosclerosis [11–13]. For example, Chen and Juo [5] showed that miR-21, miR-126, miR-365, and miR-200 are related to endothelial dysfunction, while miR-26a and Let-7 are involved in the regulation of the VSMC phenotype. Moreover, Fernandez-Hernando and Baldan [14] found that miR-758 and miR-33a/b are involved in regulating the functions of macrophages. In addition, because miRNA promoter regions contain toxic response regions, miRNAs also play an important role in toxicity responses *in vivo* [15]. Fukushima et al. [16] showed that exposure to liver toxins such as acetaminophen or carbon tetrachloride in rat caused alteration in the expression of various miRNAs. Furthermore, Izzotti et al. [17] have demonstrated that cigarette smoking can cause changes in the miRNA expression profile in the lungs of rats. However, the relationships among toxic insult, CVD processes, and miRNAs were reported less.

2,3,7,8-Tetrachlorodibenzo-*p*-dioxin (TCDD) and polychlorinated biphenyls (PCBs) are persistent organic pollutants that exist as complex mixtures *in vivo* [18, 19]. In recent years, persistent organic pollutant- (POP-) induced CVD development has been reported in many studies [20–22]. For example, we have previously shown that *in vivo* coexposure of PCBs and TCDD can enhance atherosclerosis [23], but no reports have given attention to miRNA expression profiles in response to CVD induced by TCDD and PCBs. Moreover, coherent miRNA-mRNA expression changes can occur when miRNAs decrease target mRNA levels through destabilization [15, 24]. Thus, it is of great significance to identify miRNAs with differential expression from the miRNA-mRNA CVD network.

The liver not only plays a central role in the interlinked diseases of diabetes, metabolic syndrome, and CVD but is also considered as the common target organ for environmental toxins [25, 26]. Compared with other tissues, a large number of miRNAs are located within liver cells [27]. Several studies have revealed that liver miRNAs play a pivotal role in controlling the gene expression of CVD [28, 29]. For example, Goedeke et al. found miR-148a to be a key regulator of hepatic LDL-C clearance, through the direct modulation of LDLR expression. Furthermore, liver miRNAs could be considered as hepatocyte-derived extracellular vesicles that promote endothelial inflammation and atherogenesis [30]. In our previous study, we have shown that *in vivo* coexposure of PCBs (55 mg/kg body weight) and TCDD (15 *μ*g/kg body weight) can enhance atherosclerosis [23]. Thus, in the present study, we sought to determine whether miRNA changes corresponding to the altered liver function in Apolipoprotein E-deficient (ApoE^−/−^) mice could be detected after exposure to TCDD and Aroclor1254. MicroRNA screen and miRNA-mRNA interaction analysis were therefore conducted to show whether these miRNAs play an important role in CVD processes induced by a combination of TCDD and PCBs.

## 2. Materials and Methods

### 2.1. Chemicals

TCDD (lot number: ER052609) was obtained from Cerilliant Corporation (Cerilliant, Round Rock, TX, USA), and Aroclor1254 was obtained from AccuStandard (AccuStandard, New Haven, CT, USA). TCDD and Aroclor1254 were dissolved in a minimum amount of DMSO (Amresco, Solon, OH, USA) (0.1%) and diluted to the required concentration in corn oil (Fulinmen, China). The final concentration of DMSO in TCDD and Aroclor1254 solutions administered to mice was <0.01%. All other chemicals were obtained from Sigma-Aldrich (St. Louis, MO, USA) and were of analytical grade or of the highest grade available, unless otherwise mentioned.

### 2.2. Mice

ApoE^−/−^ mice are a good model for simulating similar atherosclerotic pathological processes in the arteries of humans [31]. Thus, in the present study, twelve male ApoE^−/−^ mice (8 weeks old and 20-25 g in weight) were purchased from Weitong Lihua Biocompany (Beijing, China). The mice were housed in cages with a 12-12 h light-dark cycle, 22°C ambient temperature, and a relative humidity of 40-60%. All mice had ad libitum access to water and standard chow obtained from Peking University. The research presented was performed in compliance with the national standards established by the National Council of Animal's Use of China and was approved by the Institutional Animal Care and Use Committee of Peking University.

### 2.3. *In Vivo* TCDD and Aroclor1254 Coexposure

To determine the adverse effects of Aroclor1254 and TCDD on miRNA and mRNA profiles in the liver of ApoE^−/−^ mice, per the previously study [32], by using the complete randomization method, 12 animals were randomly assigned to the following two groups: (1) control group (corn oil, 0.4 ml i.p.); (2) TCDD and Aroclor1254 coexposure group. Mice were injected with the combined TCDD (15 *μ*g/kg b.w.) and Aroclor1254 (55 mg/kg b.w.) four times, with the first two injections in the 1^st^ week (3 days apart) and the final two injections in the 4^th^ week (3 days apart) of the 6-week study. According to previous studies, TCDD (15 *μ*g/kg b.w.) and PCB77 (49 mg/kg b.w.) led to form atherosclerotic plaques in ApoE^−/−^ mice [20, 33]. In our previous studies, we saw adhesion molecule expression in endothelial cells treated by 3.4 *μ*M PCB77 [34]. Thus, 15 *μ*g/kg b.w. of TCDD and 55 mg/kg b.w. of Aroclor1254 were chosen for the present study. This amount of POPs was based on levels that are usually found in humans after acute exposure. After 6 weeks, ApoE^−/−^ mice were fasted overnight and then anesthetized for tissue and plasma collection. The livers were excised, rinsed with phosphate-buffered saline (PBS, pH 7.4), and weighed. A portion of each liver sample was stored at -80°C until analysis. The entire aorta from the heart was removed and rinsed with PBS.

### 2.4. Oil Red O Staining

The heart tissues from ApoE^−/−^ mice in each group were frozen in optimal cutting temperature (OCT) compound, and then, Oil Red O staining was performed. Next, the atherosclerotic lesions were observed under a dissecting Zeiss AxioSkop 40 microscope (Zeiss, Germany). Finally, using ImageJ 1.44 software, mean lesion area was calculated from the digital pictures which are acquired by 9 to 12 serial sections from each mouse and quantified as described previously [23].

### 2.5. RNA Isolation

Total RNA or miRNA was isolated from the same liver and heart samples of each animal (control: *n* = 6, TCDD+Aroclor1254: *n* = 6). Total RNA was isolated using Trizol reagent (Invitrogen, Carlsbad, CA, USA), and miRNA was isolated using a miRNA Isolation Kit (Ambion, Austin, TX, USA) according to the manufacturer's instructions, respectively. The mRNA was purified with RNeasy Kit (Qiagen, Hilden, Germany), and the quantities and qualities of the mRNA were determined by 1% formaldehyde denaturing gel electrophoresis. The samples with bright bands of ribosomal 28S and 18S RNA in a ratio > 1.5 : 1 were used for microarray analysis. The quality of miRNA was determined using an Agilent Small RNA Kit according to the manufacturer's protocol. Both total RNA and miRNA samples were also quantified on a NanoDrop ND-2000 (Thermo Fisher Scientific, Waltham, MA, USA).

### 2.6. Microarray Experiments

mRNA microarrays were conducted as previously described [35]. The miRNA microarrays were performed using the Agilent Mouse miRNA array (8 × 60 K format, V18.0, Agilent Technologies, Inc.) at CapitalBio Corporation (Beijing, China) with each array containing probes for 1079 mature mouse miRNAs and 47 virus miRNAs. Information for the entire probe set is based on Sanger miRBase R18.0. Each miRNA was detected by probes repeated 30 times. miRNA microarray experiments were then conducted in accordance with the following description. Briefly, the miRNAs were labeled using the Agilent miRNA labeling reagent. Total RNA was dephosphorylated and ligated with pCp-Cy3, and the labeled RNA was purified and hybridized to miRNA arrays. Images were scanned with the Agilent microarray scanner (Agilent) and then analyzed using Agilent feature extraction software version 10.10.

The miRNA array data were analyzed for data summarization, normalization, and quality control using GeneSpring software V12 (Agilent). The default 90th percentile normalization method was performed for data preprocessing. To select the differentially expressed genes, we used threshold values of ≥1.5 and ≤−1.5-fold change and a Benjamini-Hochberg corrected *p* value of 0.05.

### 2.7. Real-Time Quantitative PCR

Two micrograms of total RNA was used to create single-stranded cDNA using the miRNA First-Strand cDNA synthesis kit (Tiangen, Beijing, China). The forward miRNA primer was synthesized according to the sequence generated by miRBase database (http://www.mirbase.org/). The universal reverse primer was purchased from Tiangen (Beijing, China). PCR was performed using GoTaq® qPCR Master Mix (Vazyme Biotech Co., Ltd., Nanjing, China) according to the manufacturer's instructions. Real-time PCR was performed using CFX96 Touch System (Bio-Rad, Hercules, CA, USA). PCR thermocycling conditions were 95°C for 10 min, followed by 40 cycles of 95°C for 30 s, 60°C for 30 s, and 72°C for 30 s. A melting curve program was used, and continuous fluorescence measurements were taken. The results are reported as the relative expression after normalization by *β*-actin, using the 2^−*ΔΔ*^Ct method. Significant differences were determined using Student's *t*-test and considered significant when *p* < 0.05. mRNA and miRNA primers are listed in Tables 1 and 2, respectively.

### 2.8. Analysis of miRNAs and Their Association with Various Diseases and Networks

For heat map generation and analysis of miRNA expression, we selected miRNAs that were up- or downregulated more than 1.5-fold in ApoE^−/−^ mice exposed to TCDD and Aroclor1254 by using MultiExperiment Viewer software (version 4.6.0, Agilent Technologies). Next, the selected miRNAs were analyzed for their role in regulating the expression of various genes and networks using Ingenuity Pathway Analysis (IPA) software and database (version 15, Ingenuity Systems Inc., CA, USA).

### 2.9. miRNA-mRNA Target Interactions after TCDD and Aroclor1254 Exposure

This process is based on two principles. The first principle, which is widely accepted and experimentally validated, is the assumption that the expression of a given miRNA is inversely correlated with the expression of its mRNA targets [24, 36]. The second principle is that we select enriched gene sets from the mRNA microarray experiment using IPA. Note that other proposed pathway-centric methodologies can be substituted for IPA in this step [37]. The rationale for this approach is that biological processes result from coordinated activation of coherent sets of genes known as modules [36, 38]. The final step of this procedure involves systematic linkage of differentially expressed miRNAs to their corresponding pathways based on the presence of predicted target genes within enriched modules. A genetic pathway was constructed for the enriched “atherosclerosis signaling pathway,” and a network was constructed for the enriched “cardiovascular system development and function” module based on experimentally verified gene product interactions derived from IPA knowledge bases.

### 2.10. Statistical Analysis

Data are depicted as means ± SEM. Statistical analyses were performed using GraphPad Prism software (GraphPad Software, San Diego, CA, USA). Single comparisons were performed using unpaired Student's *t*-test with *p* ≤ 0.05 considered as significant.

## 3. Results

### 3.1. Increased Atherosclerosis and Altered Gene Expression Profile in ApoE^−/−^ Mice after Exposure to TCDD and Aroclor1254

**Figure 1:**
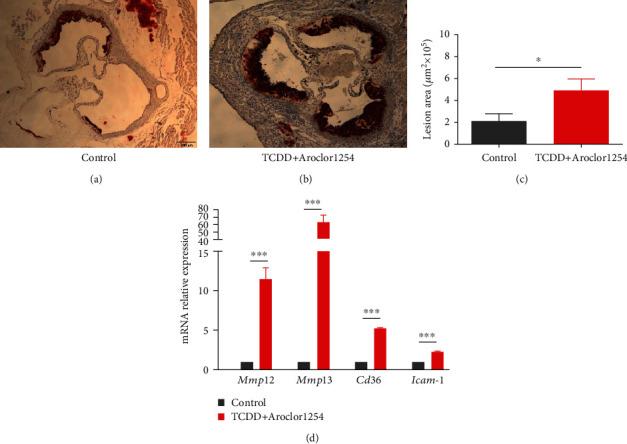
TCDD and Aroclor1254 coexposure promoted the formation of atherosclerotic lesions. Lesions of the aortic valve lipids in the (a) control and (b) TCDD+Aroclor1254 groups were visualized by Oil Red O staining (representative cross sections, magnification ×40). (c) Values are the means of the atherosclerotic lesion from individual mouse. (d) Effect of TCDD and Aroclor1254 coexposure on the mRNA expression of genes regulating atherogenesis in ApoE^−/−^ mice by RT-PCR. Data are expressed as means ± SEM (*n* = 6). ∗*p* < 0.05 and ∗∗∗*p* < 0.001, when compared between different groups.

After 6 weeks of administration, the results of Oil Red O showed that TCDD and Aroclor1254 coexposure significantly increased (4.915 ± 2.5 × 10^5^ *μ*m^2^ in the TCDD and Aroclor1254 coexposure group compared with 2.121 ± 1.67 × 10^5^ *μ*m^2^ in the control group, 2.31-fold, *p* < 0.05) the atherosclerotic lesion compared to the control group (Figures 1(a)–1(c)). In addition, we investigated the changes in atherogenesis-related genes in response to coexposure to TCDD and Aroclor1254 in the heart tissue. As shown in Figure 1(d), the mRNA levels of matrix metalloproteinase-12 (*Mmp12*), matrix metalloproteinase-13 (*Mmp13*), *CD36*, and intercellular cell adhesion molecule-1 (*Icam-1)* were significantly upregulated in the TCDD and Aroclor1254 coexposure group, relative to the control (increased by 11.45-fold, 63.83-fold, 5.25-fold, and 2.25-fold, respectively, *p* < 0.001).

**Figure 2:**
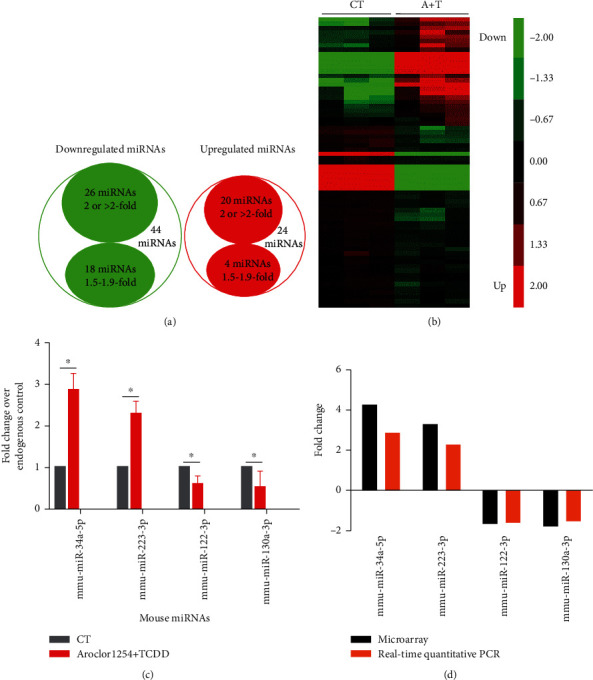
Differentially expressed miRNAs in the liver of ApoE^−/−^ mice after exposure to TCDD and Aroclor1254 and validation by RT-PCR. (a) Venn diagram showed the downregulated miRNAs (green circle) and upregulated miRNAs (red circle) after TCDD and Aroclor1254 coexposure when compared to vehicle. (b) Heat map showed changes in miRNA expression profile in the liver after coexposure to TCDD and Aroclor1254. The expression pattern (green to red) represents the spectrum of downregulated to upregulated expression pattern of miRNAs. (c) The expression profile of the miRNAs in the liver was determined by real-time PCR. Data are depicted as mean ± SEM from *n* = 6 mice/group; ∗*p* < 0.05 as compared between different groups. (d) The comparison between qPCR results and microarray data is depicted.

Based on the microarrays, a total of 68 miRNAs with fold change > 1.5 (*p* < 0.05) and 46 miRNAs with fold change > 2 (*p* < 0.05) were found in ApoE^−/−^ mice exposed to TCDD and Aroclor1254 compared with mice in the control group (Figure 2(a)). Additionally, compared with the control group, 1312 mRNAs were found to be differentially expressed (fold changes ≥ 2; *p* ≤ 0.05) in the Aroclor1254 and TCDD coexposure group (data not shown) [35]. The 68 differentially expressed miRNAs were then analyzed with cluster software. A similarity measure of miRNAs in the two groups was done using the Half Square Euclidean Distance method as previously described [37]. The visualization of the miRNA cluster analysis based on similarity between groups is shown as a dendrogram (heat map, Figure 2(b)), and miRNA expression is reflected in the range of −2 to +2 (Figure 2(b)). These data demonstrated that the Aroclor1254 and TCDD coexposure significantly altered the expression profile of genes in the liver of ApoE^−/−^ mice.

### 3.2. Validation of miRNA Expression by Real-Time PCR

To validate the miRNA array data, 4 differentially expressed miRNAs (upregulated miRs: miR-34a and miR-223; downregulated miRs: miR-122 and miR-130a) were chosen for real-time PCR analysis randomly. In Figures 2(c) and 2(d), the *x*-axis represents the miRNAs that were selected for validation, while the *y*-axis represents the fold change in miRNA expression. Within this figure, the upward bar chart indicates upregulated miRNA expression, while the downward bar chart indicates downregulated miRNA expression. Results showed upregulated expressions of miR-34a and miR-223 in the livers treated with TCDD and Aroclor1254 when compared with control-treated livers (Figures 2(c) and 2(d)). Similarly, we observed the downregulation of miR-122 and miR-130a in TCDD- and Aroclor1254-cotreated ApoE^−/−^ mice when compared with controls (Figures 2(c) and 2(d)). Thus, the real-time PCR data validated the expression profiles obtained from the arrays.

### 3.3. Dysregulation of miRNAs and mRNAs Involved in Cardiovascular Disease Pathways after TCDD and Aroclor1254 Exposure

**Figure 3:**
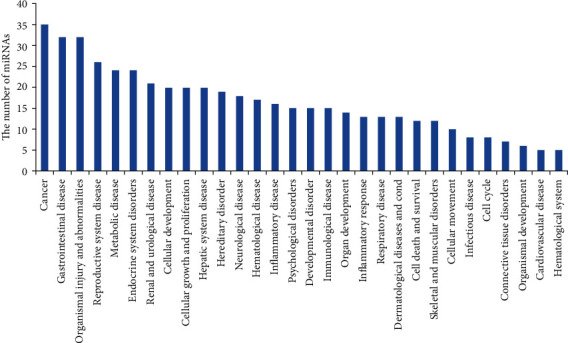
TCDD and Aroclor1254 co-exposure-regulated miRNAs and their association with diseases. TCDD and Aroclor1254 co-exposure-induced up- or downregulated (more than 1.5-fold change) miRNAs were analyzed using IPA software and the database (Ingenuity Systems Inc.). The data presented in the graph were various pathways regulated by TCDD- and Aroclor1254-coinduced miRNAs. The *y*-axis represented the number of miRNAs involved in pathways.

To further analyze the functions of differentially expressed miRNAs found in the liver of ApoE^−/−^ mice after TCDD and Aroclor1254 coexposure treatment, we used the IPA software and database to analyze the selected 68 miRNAs. The analysis revealed that there were as many as 20 pathways that may be affected by the dysregulated miRNAs (Figure 3 and Supplementary Table 1). There were 35 miRNAs involved in cancer, 24 in metabolic disease, 20 in cellular growth and proliferation, and 17 in hematological disease (Figure 3). Similarly, as shown in Figure 3, several differentially expressed miRNAs were involved in other pathways such as inflammatory disease (16 miRNAs), immunological disease (15 miRNAs), inflammatory response (13 miRNAs), cell death and survival (12 miRNAs), cellular movement (10 miRNAs), and cell cycle (8 miRNAs). Remarkably, there were five miRNAs involved in cardiovascular disease, including miR-16-5p, miR-30c-5p, miR-181a-5p, miR-26a-5p, and miR-130a-3p (data not shown).

**Figure 4:**
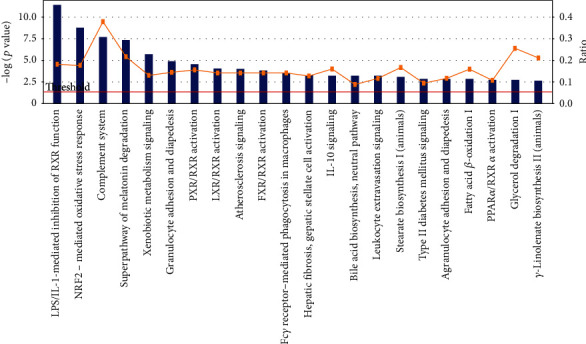
Significant canonical pathways that responded to TCDD and Aroclor1254 coexposure according to IPA software analysis. Up- and downregulated (1312 genes, fold change > 2, *p* < 0.05) mRNAs were analyzed using IPA software. The graph showed the various pathways regulated by coexposure to TCDD and Aroclor1254. The *p* value for each pathway was indicated by the line and is expressed as −1 times the log of the *p* value. The bar represented the ratio of the number of genes differentially expressed in a given pathway divided by the total number of genes that make up that pathway.

Similarly, 1312 differentially expressed mRNAs were analyzed using IPA software. As shown in Figure 4, there were more than 20 pathways that may be affected by the dysregulation of mRNAs. Figure 4 presented the top 22 pathways according to the canonical pathways in IPA. The prominent passage regulation occurred in LPS/IL-1-mediated inhibition of RXR function, IL-10 signaling, NRF2-mediated oxidative stress response, and atherosclerosis signaling.

### 3.4. miRNA-mRNA Interactions Participate in Cardiovascular Disease

**Figure 5:**
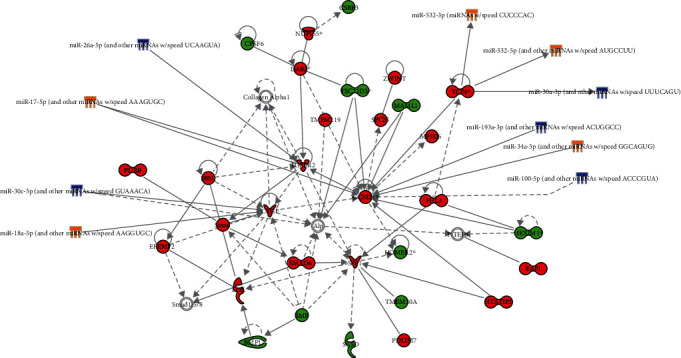
MicroRNA-mRNA interaction related to the cardiovascular system development and function network. miRNAs were used to link mRNA network related with cardiovascular system development and function to demonstrate the regulation role through IPA. Green and blue icons meant the downregulated mRNAs and miRNAs, respectively. Red and yellow icons meant the upregulated mRNAs and miRNAs, respectively. The solid arrow between miRNA and mRNAs denoted the interaction between miRNAs and mRNAs. Significantly upregulated or downregulated mRNAs: fold change > 2 (*p* value < 0.05) or fold change < 2 (*p* value < 0.05). Significantly upregulated genes or downregulated miRNAs: fold change > 1.5 (*p* value < 0.05) or fold change < 1.5 (*p* value < 0.05), respectively.

**Figure 6:**
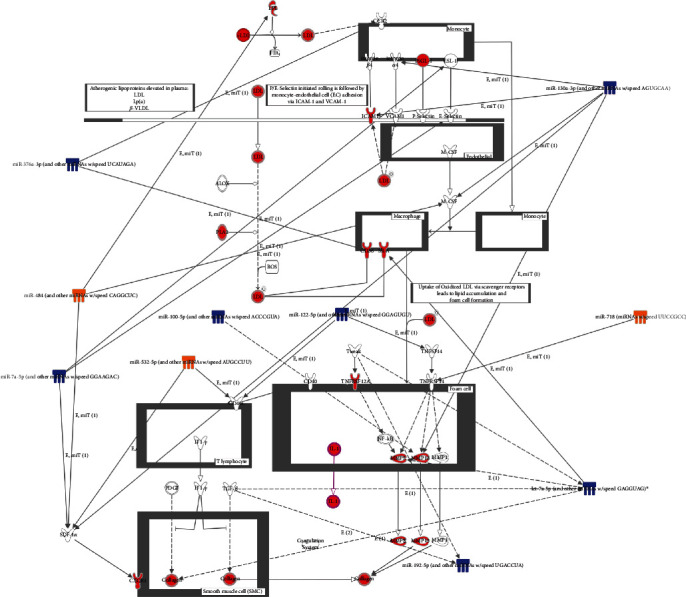
MicroRNA-mRNA networks in the atherosclerosis signaling pathway after TCDD and Aroclor1254 coexposure in ApoE^−/−^ mice. The atherosclerosis signaling pathway was significantly altered after treatment with TCDD and Aroclor1254. The mRNAs with red meant altered genes after treatment. The solid arrow between miRNA and mRNAs denoted the interaction between miRNAs and mRNAs. Significantly upregulated or downregulated mRNAs: fold change > 2 (*p* value < 0.05) or fold change < 2 (*p* value < 0.05). Significantly upregulated genes or downregulated miRNAs: fold change > 1.5 (*p* value < 0.05) or fold change < 1.5 (*p* value < 0.05), respectively.

mRNA network analysis suggested that TCDD and Aroclor1254 coexposure could change cardiovascular system development and function network and atherosclerosis signaling pathway (Supplementary Table 2). To evaluate the miRNAs which participated in cardiovascular disease induced by TCDD and Aroclor1254 coexposure, a miRNA-mRNA network approach was applied. We used the differentially expressed miRNAs and mRNAs that participated in TCDD- and Aroclor1254-responsive networks and pathways as input data, and the IPA software output is shown in Figures 5 and 6.

As shown in Figure 5, the network for cardiovascular system development and function revealed the participation of miRNAs, such as miR-26a-5p, miR-17-5p, miR-30c-5p, miR-18-5P, miR-532-3P, miR-532-5p, miR-30a-3p, miR-193a-3p, miR-34a-5p, and miR-100-5p, and mRNAs including cyclin D1 (*CCND1*), transcription factor 4 (*TCF4*), connective tissue growth factor (*CTGF*), and transforming growth factor, beta receptor II (*TGFBR2*).  Figure 6 presents the effects of Aroclor1254 and TCDD coexposure on miRNAs and mRNAs associated with the atherosclerosis signaling pathway. We can see from this figure that there are six kinds of cell (monocyte, endothelial cell, macrophage, foam cell, T lymphocyte, and smooth muscle cell) in ApoE^−/−^ mice that are associated with the atherosclerosis induced by Aroclor1254 and TCDD coexposure. miR-376a-3p, miR-484, miR-7a-5p, miR-532-5p, miR-130a-3p, miR-718, miR-122-5p, and miR-192-5p showed interactions with mRNAs that participated in atherosclerosis signaling pathways.

## 4. Discussion

The mechanism of TCDD- and PCB-induced atherosclerosis has been well characterized by many studies [21, 23, 39, 40]. Emerging data continue to demonstrate the participation of miRNAs in atherosclerosis [41–43] and in biological responses induced by environmental pollutants [44–46]. However, there have been no previous reports on miRNA expression in TCDD- and PCB-induced atherosclerosis. In our previous study, we found that TCDD and PCB coexposure in ApoE^−/−^ mice enhanced atherosclerosis development [23]. Moreover, a growing body of evidence has shown that individual exposure to TCDD or PCBs *in vivo* or *in vitro* models caused liver disease while enhancing atherosclerosis [33, 47]. Importantly, liver disease activates the expression of inflammatory factors and oxidative stress in the development of the course, further stimulating the formation of atherosclerosis [48]. Therefore, exploring the disease-specific miRNA-mRNA networks in the liver can serve as a novel mechanism for TCDD- and PCB-induced atherosclerosis in an *in vivo* animal model.

The cluster analysis data of miRNAs showed that TCDD and Aroclor1254 treatment caused significant changes in miRNA expression profiles in the livers of ApoE^−/−^ mice compared with livers of control-treated mice. Of the miRNAs screened, the expressions of 68 miRNAs were changed by more than 1.5-fold and those of 46 miRNAs were altered twofold or more. We further validated the expression profile of selected miRNAs by performing real-time PCR. All the miRNAs that we analyzed by real-time PCR corroborated the data obtained from the miRNA array analysis. The IPA pathway analysis revealed that there are five miRNAs, miR-16-5p, miR-30c-5p, miR-181a-5p, miR-26a-5p, and miR-130a-3p, that play a role in the cardiovascular disease induced by TCDD and Aroclor1254 coexposure. Moreover, the mRNA pathway analysis indicated that the atherosclerosis signaling pathway was significantly activated by TCDD and Aroclor1254 exposure.

It is widely accepted that expression changes in a small number of miRNAs have strong effects on mRNA levels. Therefore, to further determine which miRNAs regulate the atherosclerosis process, we investigated miRNAs and mRNAs that may be involved in miRNA-mRNA interactions and associated with the cardiovascular system development and function network and the atherosclerosis pathway.

### 4.1. Cardiovascular System Development and Function Network

Some of the dysregulated miRNAs could interact with multiple CVD-related mRNAs, thus playing a prominent role in the cardiovascular system development and function network. In the present study, we found that the downregulation of miRNA-26a-5p was associated with the upregulation of *TGFBR2* in the liver of ApoE^−^/^−^ mice after coexposure to TCDD and Aroclor1254. *TGFBR2* is one of CVD development effectors, which plays an essential role in vascular development [49]. Previous study showed that selective deletion of *TGFBR2* in smooth muscle caused the vascular deformities and embryonic lethality [50]. Thus, we hypothesized that miRNA-26a-5p may play an important role in the regulation of CVD induced by TCDD and Aroclor1254 coexposure treatments by regulating the expression of its target gene *TGFBR2*. Additionally, *TGFBR2* can also regulate cyclin-dependent kinase (CDK) activity by interacting with *CCND1*, thus playing an important role in cardiovascular disease [51, 52]. In this study, the cardiovascular system development and function network showed that the expression of *CCND1* was upregulated by miRNA-193a-3p [53]. Moreover, the interactions between miRNA-30c-5p and *CTGF* were also detected [54]. It has been reported that the mRNA level of *CTGF* was elevated in the aorta of diabetic ApoE^−/−^ mice, which was an important factor in the vascular ECM accumulation [55]. These gene expression changes together suggested that miRNA-26a-5p, miRNA-193a-3p, and miRNA-30c-5p may play important roles in the promotion of the CVD response following TCDD and Aroclor1254 exposure.

### 4.2. Atherosclerosis Pathway

Atherosclerosis is a major risk factor for CVD and is characterized by the accumulation of lipids and fibrous elements in the large arteries [56]. In this study, atherosclerosis pathway analysis showed that VSMCs, lymphocytes, endothelial cells, monolayer cells, macrophage, and foam cells are critical players in the atherosclerosis pathway induced by TCDD and Aroclor1254 coexposure in ApoE^−/−^ mice. There are several miRNAs and important genes together with validated and/or predicted microRNA-mRNA interactions involved in the atherosclerosis pathway. An important endothelial cell mRNA is *ICAM-1*, which is a possible target of miR-130a-3p. *ICAM-1*, as an adhesion molecule of the immunoglobulin superfamily, is critically involved in both cancer metastasis and atherogenesis. There are several pieces of evidence demonstrating that PCBs induce *ICAM-1* expression. For example, PCB104 exposure in human vessel endothelial cells could upregulate the level of ICAM-1 [57], while exposure to PCB153 not only increased the *ICAM-1* expression but also elevated adhesion of leukocytes to brain endothelial cells [58]. In the present study, our gene chip analysis showed that TCDD and PCB coexposure could upregulate the mRNA level of *ICAM-1* and that *ICAM-1* was regulated by miR-130a-3p. Notably, in a previous study, miR-130a was suggested to play an important role in maintaining normal endothelial progenitor cell (EPC) function, and dysfunction of EPCs contributes to diabetic vascular disease [59]. Other atherosclerosis risk factors, such as *Mmp13*, *M-CSF*, *sdf-1α*, and *Integrinβ2*, are also targets of miR-130a-3p possibly; however, only *Mmp13* and *ICAM-1* were upregulated in accordance with downregulated miR-130a-3p. Another important player involved in the macrophage cell functional regulation is *CD36*, and its mRNA is a possible target of miR-376a-3p. *CD36* is a lipid scavenger receptor located on the surface of macrophages that can bind oxidized low-density lipid from plasma. *CD36* can be induced by TCDD [60] and PCBs [61]. It has been reported that *CD36* is a trigger for the inflammation response and influences the development of foam cells to activate atherosclerosis [62]. Therefore, miR-376a-3p interaction with *CD36* may be an important atherosclerotic mechanism induced by TCDD and PCBs. Although other miRNA-mRNA interactions were displayed in the atherosclerosis pathway, the changes in mRNA levels were not consistent with miRNA regulation. Overall, those results strengthened the claim that miRNAs participate in atherosclerosis pathway regulation and potentially have roles in CVD processes induced by TCDD and PCBs.

## 5. Conclusion

In summary, we have detected and validated the expression levels of mRNAs and miRNAs after TCDD and PCB coexposure in ApoE^−/−^ mice. Our results suggested that miRNAs played an important role in CVD processes, such as the CVD development and function network and the atherosclerosis pathway. Furthermore, this study presented a possible miRNA-mRNA network involved in these pathways, thereby providing new insights into the mechanism of CVD in response to TCDD and PCB coexposure. Additionally, our findings established a basis for the development of combined miRNA- and mRNA-based approaches for predicting the development of CVD in response to various compounds.

## Figures and Tables

**Table 1:** Real-time PCR primers to measure mRNA expression levels.

Gene symbol	Forward sequence (5′-3′)	Reverse sequence (5′-3′)
*Mmp12*	CTCGAGGAAGCTTCCTGGGAGTCCAG	CTCGAGCCCTGAGCATAGAGTGAATATG
*Mmp13*	CTCGAGCATGCTTCCTGATGATGACGTT	CTCGAGCCCCACCCCATACATCTGAAA
*CD36*	TGGCTAAATGAGACTGGGAC	TCACCACTCCAATCCCAAGTA
*Icam-1*	GTCGAAGGTGGTTCTTCTGAGC	TCCGTCTGCAGGTCATCTTAGG
*β*-Actin	AGGCCCAGAGCAAGAGAGGTA	GGGGTGTTGAAGGTCTCAAACA

**Table 2:** Real-time PCR primers to measure miRNA expression levels.

miRNABase	Target sequences	Tiangen Cat. No.
mmu-miR-122-5p	TGGAGTGTGACAATGGTGTTTGT	N2120
mmu-miR-223-3p	TGTCAGTTTGTCAAATACCCCA	N2120
mmu-miR-34a-5p	TGGCAGTGTCTTAGCTGGTTGT	N2120
mmu-miR-130a-3p	CAGTGCAATGTTAAAAGGGCAT	N2120

## Data Availability

The data used to support the findings of this study are available from the corresponding author upon request.

## Supplementary Materials

Supplementary Materials Two tables were contained in our supplementary material file; the contents of the two tables were TCDD and Aroclor1254 co-exposure-regulated miRNAs and their association with diseases and modulation of component expression of the atherosclerosis signaling pathway in ApoE^−/−^ mice coexposed to TCDD and PCBs.
