# Supplementary material for: 2,3,7,8-Tetrachlorodibenzo-p-dioxin (TCDD) and Polychlorinated Biphenyl Coexposure Alters the Expression Profile of MicroRNAs in the Liver Associated with Atherosclerosis
Source: Biomed Res Int. 2020 Aug 12;2020:2652756. doi: 10.1155/2020/2652756 (PMC7443005; doi:10.1155/2020/2652756)
Supplement: Supplementary Materials — Two tables were contained in our supplementary material file; the contents of the two tables were TCDD and Aroclor1254 co-exposure-regulated miRNAs and their association with diseases and modulation of component expression of the atherosclerosis signaling pathway in ApoE−/− mice coexposed to TCDD and PCBs. [file 2652756.f1.docx]

Supplemental information to:

**2, 3, 7, 8 - tetrachlorodibenzo - *p* - dioxin (TCDD) and polychlorinated biphenyls co-exposure alter the expression profile of microRNAs in liver associated with atherosclerosis**

Qiuli Shan^1,2*^, Fan Qu^1^, Ningning Chen^1^

^1^ College of Biological Science and Technology, University of Jinan, Jinan 250022, China

^2^ State Key Laboratory of Environmental Chemistry and Eco-Toxicology, Research Center for Eco-Environmental Sciences, Chinese Academy of Sciences, Beijing 100085, China

Correspondence should be addressed to Qiuli Shan; [bio_shanql@ujn.edu.cn](mailto:bio_shanql@ujn.edu.cn;)

**Content**

**Supplementary Table 1**

**Supplementary Table 2**

**Supplementary Table 1** TCDD and Aroclor1254 co-exposure-regulated miRNAs and their association with diseases

| Category | *p*-Value | Number |
| --- | --- | --- |
| Cancer | 1.56E-23-4.42E-02 | 35 |
| Gastrointestinal Disease | 5.03E-22-2.45E-02 | 32 |
| Organismal Injury and Abnormalities | 4.81E-20-2.95E-02 | 32 |
| Reproductive System Disease | 4.81E-20-2.81E-02 | 26 |
| Metabolic Disease | 4.08E-11-1.11E-03 | 24 |
| Endocrine System Disorders | 1.27E-15-6.61E-04 | 24 |
| Renal and Urological Disease | 4.78E-11-1.11E-03 | 21 |
| Cellular Development | 3.67E-08-4.85E-02 | 20 |
| Cellular Growth and Proliferation | 3.67E-08-4.85E-02 | 20 |
| Hepatic System Disease | 1.22E-16-2.45E-02 | 20 |
| Hereditary Disorder | 2.67E-10-2.23E-02 | 19 |
| Neurological Disease | 4.08E-11-2.01E-02 | 18 |
| Hematological Disease | 1.56E-23-3.92E-02 | 17 |
| Inflammatory Disease | 4.78E-11-4.42E-02 | 16 |
| Psychological Disorders | 4.08E-11-4.37E-04 | 15 |
| Developmental Disorder | 2.67E-10-3.11E-02 | 15 |
| Immunological Disease | 1.56E-23-3.92E-02 | 15 |
| Organ Development | 5.90E-08-1.12E-02 | 14 |
| Inflammatory Response | 4.78E-11-2.45E-02 | 13 |
| Respiratory Disease | 3.33E-09-2.45E-02 | 13 |
| Dermatological Diseases and Cond | 3.10E-08-1.57E-02 | 13 |
| Cell Death and Survival | 3.05E-05-4.80E-02 | 12 |
| Skeletal and Muscular Disorders | 1.22E-06-3.11E-02 | 12 |
| Cellular Movement | 9.91E-06-3.98E-02 | 10 |
| Infectious Disease | 5.85E-07-1.57E-02 | 8 |
| Cell Cycle | 1.38E-05-3.77E-02 | 8 |
| Connective Issue Disorders | 5.97E-09-7.63E-08 | 7 |
| Organismal Development | 5.67E-12-1.12E-02 | 6 |
| Cardiovascular Disease | 2.12E-07-2.95E-02 | 5 |
| Hematological System | 1.56E-03-3.98E-02 | 5 |

Number represents the number of miRNAs involved in pathways.

**Supplementary Table 2** Modulation of components expression of the atherosclerosis signaling pathway in ApoE^-/-^ mice co-exposed to TCDD and PCBs (fold change > 2; *p* < 0.05)

| Symbol | Gene ID | Fold Change | Type(s) | Entrez Gene Name |
| --- | --- | --- | --- | --- |
| *CD36* | 12491 | 5.674 | transmembrane receptor | CD36 molecule |
| *COL18A1* | 12822 | -3.291 | other | collagen, type XVIII, alpha 1 |
| *CXCR4* | 12767 | 2.809 | G-protein coupled receptor | chemokine (C-X-C motif) receptor 4 |
| *ICAM1* | 15894 | 2.244 | transmembrane receptor | intercellular adhesion molecule 1 |
| *IL1RN* | 16181 | 5.686 | cytokine | interleukin 1 receptor antagonist |
| *LPL* | 16956 | 5.906 | enzyme | lipoprotein lipase |
| *MMP9* | 17395 | 2.830 | peptidase | matrix metallopeptidase 9 |
| *MMP13* | 17386 | 41.262 | peptidase | matrix metallopeptidase 13 |
| *MSR1* | 20288 | 3.829 | transmembrane receptor | macrophage scavenger receptor 1 |
| *PLA2G7* | 27226 | 3.025 | enzyme | phospholipase A2, group VII |
| *PLA2G12A* | 66350 | 2.519 | enzyme | phospholipase A2, group XIIA |
| *TNFRSF12A* | 27279 | 3.592 | transmembrane receptor | tumor necrosis factor receptor superfamily, member 12A |
